# Shared Genetic Architecture and Causal Relationship Between Serum 25-Hydroxyvitamin D and Bone Mineral Density

**DOI:** 10.1210/clinem/dgae738

**Published:** 2024-10-21

**Authors:** Linna Sha, Li Zhang, Xunying Zhao, Rong Xiang, Xueyao Wu, Jiangbo Zhu, Jiaojiao Hou, Qin Deng, Chenjiarui Qin, Changfeng Xiao, Yang Qu, Tao Han, Jinyu Zhou, Sirui Zheng, Ting Yu, Xin Song, Bin Yang, Mengyu Fan, Xia Jiang

**Affiliations:** Department of Nutrition and Food Hygiene, West China School of Public Health and West China Fourth Hospital, Sichuan University, Chengdu 610041, Sichuan, China; Department of Epidemiology and Biostatistics, West China School of Public Health and West China Fourth Hospital, Sichuan University, Chengdu 610041, Sichuan, China; Department of Pulmonary and Critical Care Medicine, State Key Laboratory of Respiratory Health and Multimorbidity, West China Hospital, West China School of Medicine, Sichuan University, Chengdu 610041, Sichuan, China; Department of Epidemiology and Biostatistics, West China School of Public Health and West China Fourth Hospital, Sichuan University, Chengdu 610041, Sichuan, China; Department of Nutrition and Food Hygiene, West China School of Public Health and West China Fourth Hospital, Sichuan University, Chengdu 610041, Sichuan, China; Department of Epidemiology and Biostatistics, West China School of Public Health and West China Fourth Hospital, Sichuan University, Chengdu 610041, Sichuan, China; Department of Nutrition and Food Hygiene, West China School of Public Health and West China Fourth Hospital, Sichuan University, Chengdu 610041, Sichuan, China; Department of Epidemiology and Biostatistics, West China School of Public Health and West China Fourth Hospital, Sichuan University, Chengdu 610041, Sichuan, China; Department of Nutrition and Food Hygiene, West China School of Public Health and West China Fourth Hospital, Sichuan University, Chengdu 610041, Sichuan, China; Department of Nutrition and Food Hygiene, West China School of Public Health and West China Fourth Hospital, Sichuan University, Chengdu 610041, Sichuan, China; Department of Epidemiology and Biostatistics, West China School of Public Health and West China Fourth Hospital, Sichuan University, Chengdu 610041, Sichuan, China; Department of Epidemiology and Biostatistics, West China School of Public Health and West China Fourth Hospital, Sichuan University, Chengdu 610041, Sichuan, China; Department of Nutrition and Food Hygiene, West China School of Public Health and West China Fourth Hospital, Sichuan University, Chengdu 610041, Sichuan, China; Department of Nutrition and Food Hygiene, West China School of Public Health and West China Fourth Hospital, Sichuan University, Chengdu 610041, Sichuan, China; Department of Nutrition and Food Hygiene, West China School of Public Health and West China Fourth Hospital, Sichuan University, Chengdu 610041, Sichuan, China; Department of Epidemiology and Biostatistics, West China School of Public Health and West China Fourth Hospital, Sichuan University, Chengdu 610041, Sichuan, China; Department of Epidemiology and Biostatistics, West China School of Public Health and West China Fourth Hospital, Sichuan University, Chengdu 610041, Sichuan, China; Department of Nutrition and Food Hygiene, West China School of Public Health and West China Fourth Hospital, Sichuan University, Chengdu 610041, Sichuan, China; Department of Epidemiology and Biostatistics, West China School of Public Health and West China Fourth Hospital, Sichuan University, Chengdu 610041, Sichuan, China; Department of Nutrition and Food Hygiene, West China School of Public Health and West China Fourth Hospital, Sichuan University, Chengdu 610041, Sichuan, China; Department of Epidemiology and Biostatistics, West China School of Public Health and West China Fourth Hospital, Sichuan University, Chengdu 610041, Sichuan, China; Department of Clinical Neuroscience, Center for Molecular Medicine, Karolinska Institutet, Stockholm 17177, Sweden

**Keywords:** serum 25-hydroxyvitamin D, bone mineral density, causal inference, genetic correlation, pleiotropic loci

## Abstract

**Context:**

Despite the well-established regulatory role of vitamin D in maintaining bone health, little is known about the shared genetics and causality of the association between serum 25-hydroxyvitamin D (25OHD) and bone mineral density (BMD).

**Objective:**

We aimed to investigate the shared genetic architecture and causal relationship between serum 25OHD and BMD, providing insights into their underlying biological mechanisms.

**Methods:**

Leveraging individual-level data from the UK Biobank (UKB) cohort and summary-level data from the genome-wide association studies (GWASs) conducted on European individuals for serum 25OHD (N = 417 580) and estimated heel BMD (eBMD, N = 426 824), we systematically elucidated the shared genetic architecture underlying serum 25OHD and eBMD through a comprehensive genome-wide cross-trait design.

**Results:**

Despite a lack of global genetic correlation ($r_{g}\;=-0.001$; *P* = .95), a statistically significant local signal was discovered at 5p11-5q11.9. Two-sample mendelian randomization (MR) indicated no causal association in the overall population (β=.003, 95% CI, −0.04 to 0.03; *P* = .93), while positive causal effects were observed in males (β=.005, 95% CI, 0.00 to 0.01; *P* = .03) and older individuals (β=.009, 95% CI, 0.00∼0.02; *P* = .01) according to one-sample MR. A total of 49 pleiotropic single-nucleotide variations (SNVs), with 4 novel SNVs (rs1077151, rs79873740, rs12150353, and rs4760401), were identified, and a total of 95 gene-tissue pairs exhibited overlap, predominantly enriched in the nervous, digestive, exocrine/endocrine, and cardiovascular systems. Protein-protein interaction analysis identified *RPS9* and *RPL7A* as hub genes.

**Conclusion:**

This study illuminates the potential health benefits of enhancing serum 25OHD levels to mitigate the risk of osteoporosis among men and individuals older than 65 years. It also unveils a shared genetic basis between serum 25OHD and eBMD, offering valuable insights into the intricate biological pathways.

Osteoporosis, characterized by low bone mineral density (BMD) and microarchitectural deterioration, poses considerable health risks to nearly one-fifth of the global population (1). Serum 25-hydroxyvitamin D (25OHD), the major circulating form of vitamin D, plays a pivotal role in calcium and mineral homeostasis, bone modeling, and bone remodeling (2). Epidemiologically, the relationship between serum 25OHD and BMD has been extensively explored over the decades—whereas some observational studies demonstrated a plausible relationship for lower levels of serum 25OHD with a decreased BMD (3, 4), others failed to establish any association (5, 6). Clinically, the inconsistent evidence further amplifies the complexity of the underlying connection. While guidelines from the National Osteoporosis Group and American Association of Clinical Endocrinologists suggest vitamin D supplementation as a strategy to reduce the risk of osteoporosis and fracture (7), meta-analyses of clinical trials have not consistently supported this recommendation (8-10). Indeed, conventional epidemiological studies are susceptible to bias, confounding, and reverse causality, making it challenging to establish causal links.

One way to disentangle the conflicting findings is to conduct mendelian randomization (MR) studies. Five MR studies have evaluated the serum 25OHD-BMD causal relationship (Supplementary Table S1) (11). While most found no significant association (12-14), a few reported a negative effect of serum 25OHD on estimated heel BMD (eBMD) (15, 16). Nonetheless, several major issues remain to be solved. First, these studies used data from genome-wide association studies (GWASs) that were of small sample size and a restricted set of instrumental variables (IVs), thereby lacking sufficient statistical power and potentially resulting in false-negative findings (12, 13, 15). Second, existing MR studies neglected sex- and age-specific effects, despite observational studies indicating apparent sex and age disparity (4-6). Third, prospective studies have identified both higher and lower levels of serum 25OHD contributing to the susceptibility of osteoporosis, implying a nonlinear relationship (3, 17), which was not examined in the current MR studies. Last but not least, existing MR studies overlooked the adjustment of obesity (ie, body mass index [BMI]), a crucial confounder determined by observational studies (18).

In addition to MR that uncovers vertical pleiotropy (ie, the effect of genetically predicted exposure on an outcome), genetic relationship can also be reflected by horizontal pleiotropy, where a single variant is responsible for both traits. Through a direct comparison of GWAS results for serum 25OHD (19) and eBMD (the most heritable bone site) (20), several loci (ie, *TRPS1* and *ABO*) have been reported as being associated with both traits, implying an intrinsic link. Nonetheless, to the best of our knowledge, no large-scale genetic analysis has been conducted to systematically investigate the extent and nature of shared etiology underlying 25OHD and eBMD, taking into account both vertical and horizontal pleiotropy, and incorporating a range of statistical genetics methods (ie, genetic correlation analysis, MR analysis, cross-trait meta-analysis, transcriptome-wide association studies, and more) that enable an extensive interrogation.

Given the high heritability estimates both for serum 25OHD ($h^{2}=32\text{\%}$ (19)) and eBMD ($h^{2}=50∼80\text{\%}$ (20)), we conducted a comprehensive genetic analysis by adopting the genome-wide cross-trait design. We first quantified global and local genetic correlations to understand a shared genetic basis underlying serum 25OHD and eBMD. We then decomposed such a shared genetic relationship into vertical pleiotropy and horizontal pleiotropy. For the former, we performed a bidirectional 2-sample MR in the general population to quantify an overall linear effect, followed by a 1-sample MR to assess a nonlinear effect as well as a sex- and age-specific effect. For the latter, we identified pleiotropic loci and shared gene-tissue pairs through cross-trait meta-analysis and transcriptome-wide association studies (TWAS), respectively. Subsequent functional annotations were made to offer novel insights into the underlying biological mechanisms. A schematic overview of the study design is shown in Fig. 1.

**Figure 1.**
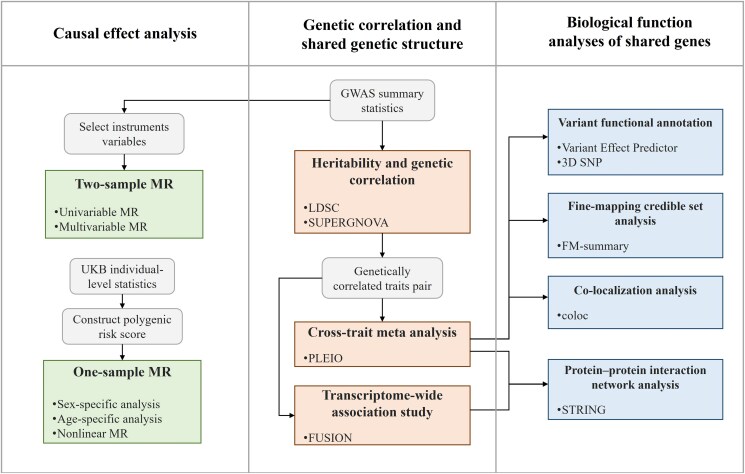
Flowchart of the overall study design. We first investigated the genetic correlation leveraging the GWASs of serum 25OHD and eBMD. We then conducted 2-sample MR and 1-sample MR to detect putative causal links between serum 25OHD and eBMD. Finally, we applied cross-trait meta-analysis and transcriptome-wide association study to identify pleiotropic loci and shared gene-tissue pairs, and further carried out a series of functional analyses to understand the potential biological mechanisms. Abbreviations: GWAS, genome-wide association study; LDSC, linkage disequilibrium score regression; MR, mendelian randomization; PLEIO, Pleiotropic Locus Exploration and Interpretation using Optimal test; STRING, Search Tool for the Retrieval of Interacting Genes/Proteins; SUPERGNOVA, SUPER GeNetic cOVariance Analyzer; UKB, UK Biobank.

## Materials and Methods

### Data Sources

Our study leveraged data from several sources, including the UK Biobank (UKB) individual-level data and the GWAS summary statistics for serum 25OHD and eBMD. Data from the UKB were employed for conducting 1-sample MR, and GWAS summary statistics for serum 25OHD and eBMD were employed for conducting 2-sample MR and the remaining components of the genome-wide cross-trait analysis.

#### UK Biobank Cohort

The UKB is a large-scale, prospective cohort study that includes deep genetic and phenotypic data from more than 500 000 participants aged 40 to 69 years (21). Participants who granted informed consent were invited to complete detailed survey questionnaires, undergo physical assessments, and provide blood samples. Following this, DNA was extracted from the blood samples and assayed using 2 novel genotyping arrays from Affymetrix Research Services Laboratory, which yielded genotype calls for 489 212 samples across 812 428 distinct markers. Serum 25OHD levels (nmol/L) were assessed through the LIAISON XL 25(OH)D assay (DiaSorin), using rank-based inverse normal transformation (RINT). eBMD (g/cm^2^) was assessed using the Sahara Clinical Bone Sonometer (Hologic Corporation) based on an ultrasound measurement of the calcaneus. We restricted the analysis to unrelated participants of White European ancestry with complete information on serum 25OHD, eBMD, and relevant covariates (N = 338 045).

#### Genome-Wide Association Study Summary Statistics of Serum 25-Hydroxyvitamin D

We used the GWAS of serum 25OHD including 417 580 European individuals aged 40 to 69 years (19). The quantification of serum 25OHD was accomplished using a chemiluminescent immunoassay in nmol/L, which captures the overall concentration of 25OHD, including 25OHD_3_ and 25OHD_2_. Independent genome-wide significant single-nucleotide variations (SNVs, formerly single-nucleotide polymorphisms [SNPs]) (*P* < 5 × 10^−8^) were identified ($R^{2}$, phenotypic variance explained by significant SNVs = 5.9%) through 10-Mb distance-based clumping with linkage disequilibrium (LD) $r^{2}<0.01$ across all variants (Supplementary Table S2) (11). We extracted relevant information of 143 serum 25OHD-associated index SNVs for IVs as well as downloaded full set summary statistics.

#### Genome-Wide Association Study Summary Statistics of Estimated Heel Bone Mineral Density

We selected the largest GWAS of eBMD including 426 824 European individuals aged 39 to 75 years, using measurements of quantitative ultrasound speed of sound and broadband ultrasound attenuation (20). The eBMD GWAS, characterized by its substantial sample size and high heritability, has established effectiveness in providing satisfactory predictive performance for osteoporosis and fractures, and has been widely applied in population-based genetic research. A total of 1103 independent genome-wide significant SNVs (*P* < 6.6 × 10^−9^) were identified ($R^{2}=25.6\text{\%}$) (Supplementary Table S3) (11). We obtained these significant eBMD-associated SNVs as IVs, along with the full set summary statistics.

Details of all contributing GWAS summary statistics are listed in Table 1.

**Table 1.** Details of genome-wide association study summary statistics

Trait	Sample size	Ethnicity	Year	Build	PMID	Measurements	Covariates
25OHD	417 580	European	2020	GRCh37	32242144	Chemiluminescent immunoassay	Age, sex, assessment mo, assessment center, supplement-intake information, genotyping batch, and first 40 ancestry principal components
eBMD	426 824	European	2018	GRCh37	28869591	Quantitative ultrasound speed of sound and broadband ultrasound attenuation	Age, sex, genotyping array, assessment center, and ancestry informative principal components 1-20
TB-BMD	66 628	86% European	2018	GRCh37	29304378	Dual-energy x-ray absorptiometry	Age, sex, weight, height, and genomic principal components as well as any additional study-specific covariates
FA-BMD	8143	European	2015	GRCh37	26367794	Dual-energy x-ray absorptiometry	Sex, age, age (2), weight
FN-BMD	32 735	European	2015	GRCh37	26367794	Dual-energy x-ray absorptiometry	Sex, age, age (2), weight
LS-BMD	28 498	European	2015	GRCh37	26367794	Dual-energy x-ray absorptiometry	Sex, age, age (2), weight

Abbreviations: 25OHD, serum 25-hydroxyvitamin D; eBMD, estimated heel bone mineral density; FA-BMD, forearm bone mineral density; FN-BMD, femoral neck bone mineral density; GEFOS, GEnetic Factors for OSteoporosis Consortium; GRCh37, Genome Reference Consortium Human Build 37; LS-BMD, lumbar spine bone mineral density; TB-BMD, total body bone mineral density; UKB, UK Biobank.

### Statistical Analysis

#### Single-nucleotide variation–based heritability and genetic correlation

We performed LD score regression (LDSC) to estimate single-trait SNV-based heritability ($h_{snp}^{2}$) and to quantify genome-wide genetic correlation ($r_{g}$) between pairs of traits (22). We used precalculated HapMap3 LD scores derived from approximately 1.2 million common- and well-imputed SNVs in European ancestry. The $r_{g}$ ranges from −1 to +1, with −1 indicating a perfect negative correlation and +1 indicating a perfect positive correlation. A *P* threshold (*P* < .05) was used to define statistical significance.

Even with a negligible global genetic correlation, there might be specific regions in the genome affecting both traits. We applied SUPERGNOVA (SUPER GeNetic cOVariance Analyzer) to estimate local genetic correlation (23). This algorithm used LDetect methods (24) to partition the whole genome into 2353 LD-independent regions with an average length of 1.6 cM and precisely quantifies the shared genetic correlation for each genomic region. A Bonferroni-corrected *P* threshold (*P* < .05/2353) was used to define statistical significance.

Both LDSC and SUPERGNOVA can provide accurate estimates of genetic correlation, even in the presence of sample overlap.

#### Two-sample mendelian randomization

Genetic correlation can be decomposed into vertical and horizontal pleiotropy. We first conducted a bidirectional 2-sample MR analysis to detect putative causal links between serum 25OHD and eBMD in the general population (vertical pleiotropy). Inverse-variance weighted (IVW) was applied as our primary approach. Complementary to IVW, MR-Egger regression, weighted median approach, and MR-PRESSO were performed to examine the robustness and consistency of results (25). The estimate was considered robust if it achieved statistical significance (*P* < .05) in IVW and maintained directional consistency in all the other 3 methods.

To validate the main model assumptions of MR, we performed several sensitivity analyses. First, we repeated IVW removing pleiotropic IVs associated with potential confounders according to the GWAS Catalog. Second, we conducted a leave-one-out analysis, excluding one IV for serum 25OHD at a time and reestimating the causal effect. We also conducted an MR Steiger test to validate the direction of causality, excluding directionally discordant SNVs and reestimating the causal effects. Third, we performed multivariable MR analysis (26) to estimate the independent causal effect taking into account the major confounder BMI. Last, we included BMDs of total body (27), forearm, femoral neck, and lumbar spine (28), derived from dual-energy x-ray absorptiometry (DXA) scanning (a modality prevalent in clinical settings), as secondary outcomes.

#### One-sample mendelian randomization

We next conducted a one-sample MR analysis to elucidate a nonlinear relationship as well as to understand a sex- and age-specific effect for 25OHD and eBMD. We constructed an unweighted polygenic risk score by combining 143 independent SNVs from the GWAS of serum 25OHD (19), for estimating genetically predicted serum 25OHD levels. Due to the overlap between the GWAS summary data and our study sample, external weights could not be applied (29).

We first conducted a linear one-sample MR to detect causal relationships between serum 25OHD and eBMD in the overall, sex-, and age-specific populations. To account for the potential influence of hormonal factors on bone health (30), we further stratified the female participants into premenopausal and postmenopausal groups. Additionally, we performed nonlinear MR for the exploration of nonlinearity. Participants were categorized into 4 subgroups based on quartiles of residual serum 25OHD levels, calculated by the observed serum 25OHD levels minus the mean-centered genetic contribution to serum 25OHD derived from IVs (31). Linear MR analyses were conducted across the overall population and each subgroup, employing the Wald ratio method (32) with adjustments for age, sex, assessment center, assessment month, vitamin D supplement, BMI, genotype array, and the top 40 genetic principal components. Subsequently, we assessed heterogeneity using the Cochran *Q* statistic and examined nonlinearity through meta-regression of MR estimates against the mean of serum 25OHD in each subgroup (31). A *P* threshold (*P* < .05) was used to define statistical significance. The analyses were conducted using the R base package and “metafor” package.

#### Cross-trait meta-analysis

We conducted a cross-trait meta-analysis using PLEIO to identify pleiotropic SNVs affecting both serum 25OHD and eBMD (33). PLEIO is a summary-statistic–based approach to map pleiotropic SNVs in a joint analysis of complex traits. This method can maximize power by adequately modeling genetic correlations and control false-positive rates by accounting for environmental correlations.

To identify independent pleiotropic SNVs, we performed the clumping function in PLINK software: –clump-p1 5e-8 –clump-p2 1e-5 –clump-r2 0.2 –clump-kb 500. Loci were defined as 1-MB regions (±500 kb) around each pleiotropic SNV. Within each locus, the SNV with the lowest *P* value was selected as the index SNV. Statistically significant pleiotropic SNVs were defined as index variants with *P*_PLEIO_ less than 5 × 10^−8^ and *P*_single-trait_ less than 1 × 10^−5^ (for both traits). Particularly, a statistically significant pleiotropic SNV satisfies the following conditions was further considered as a novel pleiotropic SNV: (1) the SNV was not driven by any single trait (5 × 10^−8^ < *P*_single-trait_ < 1 × 10^−5^); (2) the SNV was not in LD ($r^{2}<0.2$) with any previously reported genome-wide significant single-trait SNVs, and none of its neighboring SNVs (±500 kb) reached *P* less than 5 × 10^−8^ in single-trait GWAS.

Ensemble Variant Effect Predictor (https://grch37.ensembl.org/Homo_sapiens/Tools/VEP) and 3DSNP (https://omic.tech/3dsnpv2/) were used to map the linear closest genes and the 3-dimensional interacting genes for pleiotropic SNVs, respectively.

#### Fine-mapping credible-set analysis and colocalization analysis

The index SNV does not always represent the causal SNV. We further conducted a fine-mapping analysis using FM-summary (34, 35) to identify a credible set of SNVs that were 99% likely to contain the causal variants at each of the pleiotropic loci obtained from PLEIO. This Bayesian fine-mapping algorithm uses a flat prior with steepest descent approximation to map the primary signal within a given region, assuming that at least one causal variant exists (35). We extracted variants within 500 kb of each index SNV and calculated the posterior probability of each SNV being a causal variant. The credible set for each index SNV was then determined, defined as the minimum set of SNVs whose cumulative probability accounted for 99%.

We performed a colocalization analysis using coloc (36) to detect whether pleiotropic loci colocalized at the same causal variant. Coloc uses a Bayesian framework to derive 5 easily interpretable posterior probabilities relating to 5 hypotheses: H0 (no causal variant); H1 and H2 (causal variant for only 1 trait); H3 (2 causal variants), and H4 (a joint causal variant). We extracted summary statistics for variants within 500 kb of each pleiotropic index SNV and calculated the posterior probabilities under default prior settings. A locus was considered colocalized if PPH4 was greater than 0.75.

#### Transcriptome-wide association studies

We performed TWAS using FUSION by integrating GWAS summary statistics with expression weights across 49 tissues from the Genotype-Tissue Expression project (GTEx, version 8) (37). First, we performed 49 TWASs for each trait, one tissue-trait pair at a time. Then, we conducted joint/conditional tests using a summary-statistic-based method (38) to identify an independent set of gene-tissue pairs. To account for multiple comparisons, we applied Bonferroni correction across all gene-tissue pairs (293 379 for serum 25OHD and 293 494 for eBMD), with a significance threshold of *P*_Bonferroni_ < .05. Finally, we intersected single-trait TWAS results to determine shared gene-tissue pairs across traits.

#### Protein–protein interaction network analysis

We used STRING (Search Tool for the Retrieval of Interacting Genes/Proteins) (39) to construct a network of protein-protein interaction (PPI) for the genes located at pleiotropic loci identified in PLEIO and transcriptome-wide shared genes obtained from TWAS. The basic assumption posits that genes that interact directly (physically) or indirectly (functionally) may have the same or similar functions in the biological processes. The interaction scores, derived from different sources including experimentally determined interactions, database annotated information, and automated text mining knowledge, were used. PPI pairs with an interaction score greater than 0.4 were then extracted. Subsequently, the visualized network of PPI pairs was generated using Cytoscape software, and the connectivity degree of individual protein node was computed through CytoHubba plugin. Finally, we considered the top 20 genes as hub genes crucial in the PPI network, and identified hub protein nodes of the PPI network with a connectivity degree of 10 or greater.

## Results

### Single-Nucleotide Variation–based Heritability and Genetic Correlation

**Figure 2.**
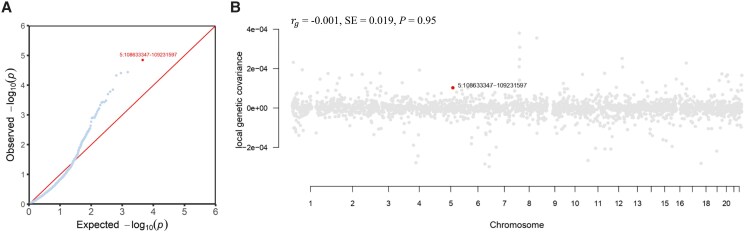
Local genetic correlation between serum 25-hydroxyvitamin D (25OHD) and estimated heel bone mineral density (eBMD). (A) Quantile-quantile (QQ) plot presents region-specific *P* values from the local genetic correlation by SUPERGNOVA. (B) Manhattan-style plot shows the estimates of local genetic covariance. Bold dots represent a genomic region contributing to a significant local genetic correlation with serum 25OHD and eBMD after multiple testing adjustments (*P* < .05/2353). $r_{g}$, genetic correlation.

Both serum 25OHD and eBMD were heritable, with an estimated $h_{snp}^{2}$ of 0.10 and 0.27, respectively. Although no statistically significant global genetic correlation was observed ($r_{g}\;=-0.001$; *P* = .95), a genomic region at 5p11-5q11.9 (chromosome 5: 108633347-109231597) was identified as contributing to a significant local genetic correlation with serum 25OHD and eBMD (Fig. 2). This genomic region harbors 1 previously reported serum 25OHD locus (*KRT18P42*) and 2 previously reported eBMD loci (*LOC285638* and *PJA2*).

### Two-Sample Mendelian Randomization

**Figure 3.**
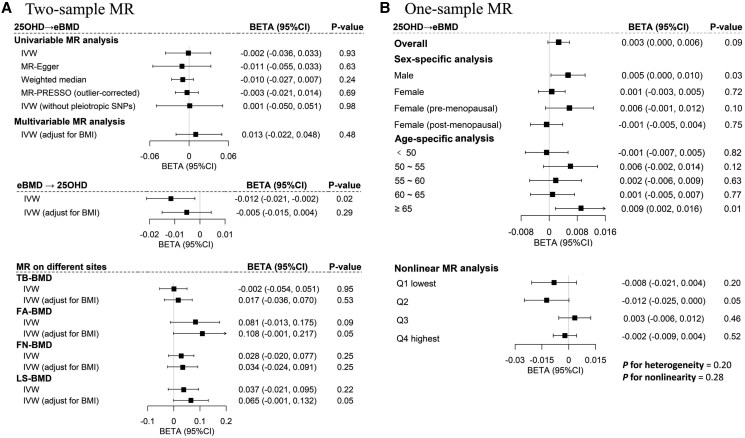
Causal relationships between serum 25-hydroxyvitamin D (25OHD) and bone mineral density. (A) Bidirectional 2-sample mendelian randomization (MR) analysis. We applied univariate MR and multivariate MR to explore bidirectional causality between serum 25OHD and estimated heel bone mineral density (eBMD). (B) One-sample MR analysis. Linear MR was conducted across the overall, sex-, and age-specific populations, followed by nonlinear MR categorizing participants into 4 subgroups based on quartiles of residual serum 25OHD levels. Abbreviations: FA-BMD, forearm bone mineral density; FN-BMD, femoral neck bone mineral density; LS-BMD, lumbar spine bone mineral density; Q, quartile; TB-BMD, total body bone mineral density.

As shown in Fig. 3A and Supplementary Table S4 (11), high serum 25OHD concentration, due to genetic predisposition across the 143 SNVs, was not associated with higher eBMD in the IVW analysis (β=−.00, 95% CI, –0.04 to 0.03; *P* = .93). This null effect remained consistent in MR-Egger regression (β=−.01, 95% CI, –0.06 to 0.03; *P* = .62), the weighted median approach (β=−.01, 95% CI, –0.03 to 0.01; *P* = .24), and MR-PRESSO (β=−.00, 95% CI, –0.02 to 0.01; *P* = .69). The sensitivity analysis yielded consistent results with the main analysis (Supplementary Tables S5-S8) (11). Moreover, no causal effect was observed for serum 25OHD on BMDs of total body, at the forearm, femoral neck, or lumbar spine. Reverse-directional MR did not reveal any association by IVW after adjusting for BMI (β=−.01, 95% CI, –0.02 to 0.00; *P* = .29).

### One-Sample Mendelian Randomization

We continued to understand the sex- and age-specific causal association as well as a nonlinear effect through one-sample MR (Fig. 3B and Supplementary Table S9 (11)). Consistent with results from 2-sample MR, genetically predicted serum 25OHD showed no causal association with eBMD in the overall population (β=.003, 95% CI, –0.00 to 0.01; *P* = .09). In the sex-specific analysis, although no statistically significant causal effect was observed in women (including both the premenopausal and postmenopausal groups), a positive causal relationship was identified among men (β=.005, 95% CI, 0.00∼0.01; *P* = .03). In the age-specific analysis, a positive causal relationship was observed among individuals aged 65 years and older (β=.009, 95% CI, 0.00∼0.02; *P* = .01). As for the nonlinear relationship, a null causal relationship across all residual serum 25OHD subgroups was observed with the simultaneous absence of heterogeneity (*P* = .20) and nonlinearity (*P* = .28).

### Cross-Trait Meta-analysis and Pleiotropic Loci

A total of 49 significant pleiotropic loci were identified (Table 2 and Supplementary Table S10 (11)). Notably, SNV rs2737252 (*P*_PLEIO_ = 8.69 × 10^−97^), the strongest pleiotropic signal, was mapped to the protein-coding gene *TRPS1*. SNV rs117363662 (*P*_PLEIO_ = 3.17 × 10^−81^) represented the second strongest pleiotropic signal, located near gene *CALCB*. SNV rs635634 (*P*_PLEIO_ = 1.07 × 10^−59^) was the third strongest pleiotropic signal, mapped to the gene *ABO*.

**Table 2.** Statistically significant pleiotropic loci between serum 25-hydroxyvitamin D and estimated heel bone mineral density identified from cross-trait meta-analysis

Index SNV	EA	OA	25OHD		eBMD		*P*_PLEIO_	Cytoband region	Linear closest genes	3D interacting genes	PPH4
			β	*P*	β	*P*					
**rs7528419** *^a^*	A	G	−.02	1.35 × 10^−16^	−.01	3.60 × 10^−9^	1.53 × 10^−29^	1p13.3	*CELSR2, PSRC1*	*CELSR2* and other 8	0.992
rs1892419	T	C	−.01	2.66 × 10^−9^	.01	2.40 × 10^−6^	8.09 × 10^−16^	1p34.2	*—*	*EDN2*	<0.001
rs7554349	C	G	.01	2.91 × 10^−6^	.02	4.10 × 10^−8^	6.71 × 10^−14^	1q43	*FMN2*	—	0.008
rs7585120	C	T	−.01	1.64 × 10^−8^	−.02	2.30 × 10^−12^	7.47 × 10^−30^	2q14.1-2q14.2	*—*	*CCDC93* and other 2	<0.001
rs62165280	G	A	−.01	4.39 × 10^−7^	−.03	2.60 × 10^−24^	8.31 × 10^−51^	2q14.2	*AC093901.1*	*INSIG2, CCDC93*	<0.001
rs6002323	T	C	.01	1.15 × 10^−6^	−.01	2.70 × 10^−10^	1.02 × 10^−18^	2q14.2	*ZC3H7B*	*ZC3H7B* and other 3	<0.001
rs111873781	C	T	−.02	6.83 × 10^−9^	−.01	5.20 × 10^−6^	4.80 × 10^−15^	3p21.31	*MST1R*	*MST1R* and other 11	<0.001
rs3774751	G	T	.01	4.42 × 10^−7^	.02	5.50 × 10^−19^	4.03 × 10^−35^	3p21.31	*MIR566, SEMA3F*	*GNAT1* and other 7	<0.001
**rs899631** *^a^*	G	T	.01	7.41 × 10^−9^	−.02	2.70 × 10^−19^	3.07 × 10^−40^	4q12	*—*	*POLR2B* and other 3	0.898
rs7661844	C	T	−.01	3.86 × 10^−6^	.01	9.80 × 10^−11^	6.03 × 10^−23^	4q21.3	*MAPK10, MIR4452*	*PTPN13, C4orf36*	0.005
**rs1471251** *^b^*	A	T	.01	4.03 × 10^−7^	−.02	1.20 × 10^−25^	2.71 × 10^−56^	4q21.3-4q22.1	*AFF1*	*AFF1* and other 2	<0.001
rs71607411	A	T	−.01	1.56 × 10^−9^	.01	1.50 × 10^−6^	6.38 × 10^−23^	4q21.3-4q22.1	*KLHL8*	*KLHL8* and other 5	<0.001
rs10020067	C	A	.01	9.86 × 10^−13^	−.01	3.50 × 10^−11^	8.66 × 10^−32^	4q22.1	*RP11-529H2.1*	*HSD17B13* and other 5	<0.001
rs965807	G	T	−.01	3.30 × 10^−6^	−.02	1.10 × 10^−16^	2.45 × 10^−33^	4q22.2-4q22.3	*RP11-363G15.2*	—	0.278
rs7746495	G	T	−.01	4.34 × 10^−6^	.01	1.70 × 10^−9^	1.01 × 10^−15^	6q25.3	*ARID1B*	—	0.348
rs904018	T	C	.01	9.34 × 10^−9^	.02	1.10 × 10^−22^	6.37 × 10^−38^	8p23.1	*C8orf49, GATA4*	*C8orf49* and other 6	<0.001
rs13261622	C	T	.01	4.68 × 10^−6^	.02	1.20 × 10^−13^	2.05 × 10^−29^	8p23.1	*—*	*PRSS55*	0.127
rs11776499	G	A	−.01	1.80 × 10^−6^	−.02	1.10 × 10^−12^	1.31 × 10^−20^	8p23.1	*MSRA*	*MSRA*	0.091
rs2737252	G	A	−.01	8.19 × 10^−6^	−.03	2.90 × 10^−55^	8.69 × 10^−97^	8p23.3	*TRPS1*	*TRPS1*	<0.001
rs17661353	G	A	−.01	4.28 × 10^−6^	.01	1.90 × 10^−8^	6.80 × 10^−15^	8p23.3	*—*	—	<0.001
**rs635634** *^a^*	C	T	.02	8.82 × 10^−13^	.03	6.80 × 10^−24^	1.07 × 10^−59^	9q34.2	*ABO*	*ABO* and other 13	0.997
rs10767659	G	T	−.01	2.14 × 10^−6^	.01	1.60 × 10^−8^	1.08 × 10^−12^	11p14.1	*BDNF, BDNF-AS*	*BDNF* and other 3	0.010
rs4597047	T	C	.02	2.15 × 10^−8^	−.02	2.50 × 10^−8^	1.09 × 10^−19^	11p15.2	*INSC*	*INSC, CALCB*	<0.001
rs61880664	A	G	.02	7.23 × 10^−11^	−.03	2.40 × 10^−21^	1.10 × 10^−44^	11p15.2	*—*	—	<0.001
rs11023763	C	T	−.02	5.85 × 10^−12^	−.01	6.70 × 10^−10^	6.32 × 10^−22^	11p15.2	*—*	—	<0.001
rs117363662	G	A	.11	5.25 × 10^−54^	−.03	1.30 × 10^−6^	3.16 × 10^−81^	11p15.2	*CALCB*	*CALCB* and other 3	<0.001
rs72872035	A	C	.06	1.37 × 10^−35^	.02	5.90 × 10^−6^	2.23 × 10^−51^	11p15.2	*CALCB*	*CALCB* and other 4	<0.001
rs1440715	A	G	.01	1.74 × 10^−6^	.02	1.40 × 10^−10^	5.24 × 10^−16^	11p15.2	*—*	—	<0.001
rs1320210	G	A	.01	7.26 × 10^−6^	−.01	3.10 × 10^−6^	1.63 × 10^−14^	11p15.2	*—*	—	<0.001
rs589030	G	C	.01	1.02 × 10^−7^	−.01	5.30 × 10^−6^	1.08 × 10^−16^	11q13.1	*PLCB3*	*PLCB3* and other 18	0.106
rs11052759	G	A	−.01	7.82 × 10^−9^	.01	3.60 × 10^−6^	5.03 × 10^−12^	12p11.1	*RNU6-400P*	*SYT10*	0.122
**rs11045856** *^a^*	T	G	−.01	2.86 × 10^−9^	.01	2.20 × 10^−6^	4.38 × 10^−17^	12p12.2-12p12.1	*SLCO1B1*	*SLCO1B1*	0.975
**rs4149056** *^a^*	T	C	.02	9.00 × 10^−14^	−.02	5.90 × 10^−8^	4.20 × 10^−30^	12p12.2-12p12.1	*RP11-125O5.2, SLCO1B1*	*SLCO1B1*	0.975
**rs4760401** *^c^*	A	G	−.01	1.24 × 10^−6^	−.01	2.60 × 10^−6^	7.28 × 10^−13^	12q22	*PLEKHG7*	*C12orf74, PLEKHG7*	0.362
rs9324063	G	T	.01	1.95 × 10^−6^	−.02	3.30 × 10^−18^	1.97 × 10^−37^	14q32.32-q32.33	*TRMT61A*	*TRMT61A* and other 6	<0.001
rs34411783	G	A	−.01	5.05 × 10^−6^	−.02	8.20 × 10^−18^	1.57 × 10^−37^	14q32.32-q32.33	*MARK3*	*MARK3* and other 4	<0.001
rs861544	G	A	.01	6.65 × 10^−7^	.01	3.40 × 10^−10^	2.04 × 10^−21^	14q32.33	*KLC1, RP11-73M18.10, RP11-73M18.7, RP11-73M18.8, XRCC3*	*KLC1* and other 4	<0.001
rs55696130	A	G	−.01	4.84 × 10^−7^	.01	1.50 × 10^−7^	1.29 × 10^−16^	14q32.33	*CTD-2134A5.3, CTD-2134A5.4*	*LINC00637* and other 6	<0.001
rs56369308	T	C	.02	2.29 × 10^−18^	−.01	3.00 × 10^−9^	7.74 × 10^−38^	15q22.31	*USP3*	*USP3* and other 4	<0.001
rs35733741	G	A	−.01	2.53 × 10^−9^	−.01	9.10 × 10^−9^	3.79 × 10^−19^	16p11.2	*FBXL19*	*FBXL19* and other 16	0.063
rs8614	C	A	.01	1.41 × 10^−7^	.01	3.30 × 10^−8^	2.82 × 10^−17^	17q11.2	*NUFIP2*	*NUFIP2* and other 4	<0.001
rs10454087	C	T	.01	8.69 × 10^−10^	−.01	1.10 × 10^−9^	4.92 × 10^−24^	17q21.2	*FAM134C*	*FAM134C* and other 13	0.274
**rs12150353** *^c^*	C	T	.01	2.80 × 10^−6^	−.01	6.80 × 10^−6^	9.04 × 10^−14^	17q21.31	*DHX8*	*DHX8* and other 5	0.002
**rs11665052** *^a^*	A	G	.01	2.77 × 10^−8^	−.02	1.20 × 10^−9^	1.34 × 10^−27^	18q21.32	*—*	*MC4R*	0.956
**rs1077151** *^c^*	A	G	−.01	6.73 × 10^−6^	.01	5.50 × 10^−8^	5.98 × 10^−15^	19p13.2	*NFIX*	*NFIX* and other 12	0.009
**rs3810242** *^a^*	G	T	−.01	5.39 × 10^−7^	.01	5.50 × 10^−7^	1.16 × 10^−13^	19q13.42	*AC012314.1, CNOT3*	*CNOT3* and other 7	0.759
**rs79873740** *^a,c^*	C	T	−.02	2.37 × 10^−7^	−.02	2.90 × 10^−6^	2.64 × 10^−14^	20q13.33	*—*	*SLC2A4RG* and other 19	0.935
rs13054331	G	A	.01	2.84 × 10^−7^	.01	4.70 × 10^−6^	3.49 × 10^−14^	22q11.22-22q11.23	*RTDR1*	*RTDR1* and other 2	0.627
**rs5770908** *^a^*	G	A	.01	2.70 × 10^−8^	.02	7.10 × 10^−19^	7.33 × 10^−29^	22q13.33	*PPP6R2*	*PPP6R2* and other 21	0.928

(*P*_PLEIO_ < 5 × 10^−8^, *P*_single-trait_ < 1 × 10^−5^).

Abbreviations: 3D, 3-dimensional; 25OHD, 25-hydroxyvitamin D; AEA, effect allele; eBMD, estimated heel bone mineral density; OA, other allele; PLEIO: Pleiotropic Locus Exploration and Interpretation using Optimal test; LD, linkage disequilibrium; SNV, single-nucleotide variation.

^*a*^SNV is probably associated with both traits and shares a single causal variant, colocalization PPH4 greater than 0.75.

^*b*^SNV has only one candidate causal SNV in the credible set (itself), with a posterior probability of 1.00.

^*c*^Novel pleiotropic SNVs (5 × 10^−8^ < *P*_single-trait_ < 1 × 10^−5^; LD *r*^2^ < 0.2).

Among these 49 pleiotropic loci, 4 were identified as novel. The most significant novel signal rs1077151 (*P*_PLEIO_ = 5.98 × 10^−15^) was mapped to *NFIX*, encoding a transcription factor related to mental and physical development. The second most significant novel signal rs79873740 (*P*_PLEIO_ = 2.65 × 10^−14^) was mapped to the transcriptional regulatory genes *SLC2A4RG* and *ZGPAT*. Both genes have been reported to be associated with breast cancer. The third most significant novel signal rs12150353 (*P*_PLEIO_ = 9.04 × 10^−14^) was located near *DHX8*, which encodes a DEAH box polypeptide. The fourth most significant novel signal rs4760401 (*P*_PLEIO_ = 7.28 × 10^−13^) was located near *PLEKHG7*, which enables guanyl-nucleotide exchange factor activity (40).

### Identification of Causal Variants and Colocalization

For each PLEIO-identified locus, we identified a 99% credible set of causal SNVs as detailed in Supplementary Table S12 (11), providing targets for downstream experimental analysis that could further confirm their functional effect and elucidate their roles in the biological process. In total, we found 1284 candidate causal SNVs across all loci shared by serum 25OHD and eBMD. Notably, at the locus of index SNV rs1471251, the 99% credible set consisted of only one candidate variant (rs1471251 itself), with a posterior probability of 1.00.

Colocalization analysis was further performed to determine whether the pleiotropic SNVs driving the association in two traits were the same or different. Nine pleiotropic loci (index SNVs: rs7528419, rs635634, rs11665052, rs5770908, rs4149056, rs11045856, rs79873740, rs899631, and rs3810242) colocalized at the same candidate SNVs (PPH4 > 0.75) (Supplementary Table S13) (11). Of interest, SNV rs635634 was the third strongest pleiotropic SNV and rs79873740 was a novel pleiotropic SNV.

### Transcriptome-wide Association Studies

Results from TWAS revealed gene-level genetic overlap between serum 25OHD and eBMD. A total of 48 TWAS-significant shared genes were identified, most of which were enriched in tissues of the nervous, digestive, exocrine/endocrine, and cardiovascular systems (Supplementary Table S14) (11). A majority of these genes (25/48) were located at pleiotropic loci identified in the PLEIO. Particularly, 6 genes were located at colocalized loci, including *CELSR2*, *PSMA5, PSRC1* at 1p13.3, *REST* at 4q12, *ABO* at 9q34.2, and *PPP6R2* at 22q13.33.

Among the 48 TWAS-significant shared genes, 31 (64.6%) were previously reported to be associated with both serum 25OHD and/or eBMD (GWAS Catalog accessed February 24, 2024). Specifically, 12 genes were previously reported to be associated with both serum 25OHD and eBMD, while 18 were associated only with eBMD, and 1 was associated only with serum 25OHD. We identified 17 (35.4%) novel genes. Although the biologic functions of several genes (*AC145124.2*, *AF131216.7*, *DEFB109D*, *ENSG00000271754.1*, *RP11-194N12.2*, *RP11-196G11.3*, *RP11-981G7.3*, and *RP11-981G7.6*) remain unclear, other novel genes are implicated in various biological processes, including cell proliferation and transformation (*FIBP* and *PTPN13*), metabolic regulation (*PIPOX* and *AGMAT*), protein degradation (*PSMA5* and *RNF123*), synapse formation and maintenance (*BSN*), involvement in the autophagy pathway (*BECN1*), and regulation of gene transcription (*SETD1A*) (40).

### Protein-Protein Interaction Network Analysis

**Figure 4.**
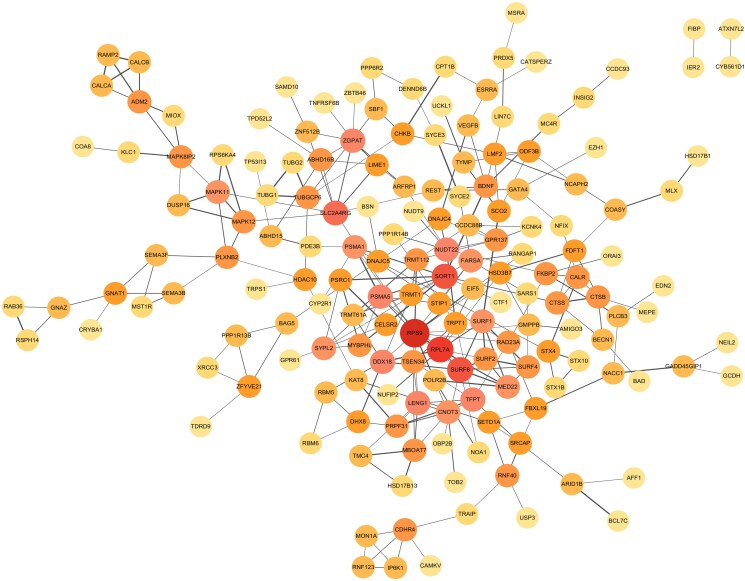
Protein-protein interaction network of shared genes between serum 25-hydroxyvitamin D (25OHD) and estimated heel bone mineral density (eBMD). We constructed protein-protein interaction network for the genes located at pleiotropic loci identified in Pleiotropic Locus Exploration and Interpretation using Optimal test (PLEIO) and transcriptome-wide shared genes obtained from transcriptome-wide association studies (TWAS). The circles represent genes, with deeper color indicating a higher connectivity degree. The lines indicate interactions among genes, with thicker lines representing higher combined scores.

The total PPI network of serum 25OHD and eBMD was constructed, consisting of 167 nodes and 271 edges (Fig. 4 and Supplementary Table S15 (11)). The highest interaction PPI pairs were *PSMA1-PSMA5*, *RPS9*-*RPL7A*, and *TUBG1-TUBGCP6*, each with an interaction score of 0.999. The top 20 hub genes of the total PPI network are shown in Supplementary Fig. S2 and Supplementary Table S16 (11). Among these, *RPS9* and *RPL7A* were identified as hub protein nodes, exhibiting the highest connectivity degrees of 16 and 10, respectively. Notably, both genes were located at colocalized loci, 19q13.42 and 9q34.2, respectively.

## Discussion

To the best of our knowledge, this is the first comprehensive genetic analysis to systematically investigate the shared genetic architecture underlying serum 25OHD and eBMD. Leveraging summary statistics from the hitherto largest GWASs, we applied a series of advanced genetic methodologies to thoroughly examine genetic correlations, causal relationships, pleiotropic loci, and shared gene-tissue pairs between serum 25OHD and eBMD. Our findings provide evidence supporting a locally shared genetic basis, suggesting shared biology between the two traits. This genetic overlap was further decomposed into both causality and pleiotropy, reflected by the sex- and age-specific (rather than overall) causal relationship demonstrated by MR, the pleiotropic loci identified in PLEIO, and the shared genes revealed by TWAS.

Despite the limited global genetic correlation between serum 25OHD and eBMD, a significant local genetic correlation was found for a specific genomic region at 5p11 to 5q11.9. Within this region, a protein-coding gene, *PJA2*, was found to downregulate Wnt/*β*-catenin signaling activity by reducing the levels of transcriptional factors *TCF/LEF1* (41). This pathway plays a critical role in maintaining bone health, orchestrating the activities of both bone-forming osteoblasts and, indirectly, bone-resorbing osteoclasts (42). These findings suggest a potential complex link underlying serum 25OHD and eBMD that deserves further investigation.

A shared genetic basis can be the result of vertical pleiotropy and/or horizontal pleiotropy. In our downstream analysis performed to explore these alternatives, we found no association of serum 25OHD with eBMD in the general population, largely in line with earlier MR studies (12-16). However, we considerably expanded on the understanding across 3 critical aspects. First, our study used more than 100 IVs derived from the serum 25OHD GWASs, which substantially improved the strength of genetic instruments and avoided the potential for false-negative findings, yielding robust effect estimates. Second, previous MR studies mainly focused on single-source outcomes. Our study included both ultrasound-derived BMD (eBMD) and DXA-derived BMDs (TB-BMD, FA-BMD, FN-BMD, LS-BMD), corroborating each other to provide solid evidence for the 25OHD-BMD relationship. Of utmost importance, our study performed a detailed one-sample MR with UKB individual-level data to facilitate the exploration of sex- and age-specific effects as well as nonlinear effects. We identified a positive causal effect of serum 25OHD on eBMD in men rather than women. This finding is consistent with previous observational studies (4-6), highlighting a more pronounced effect observed in men. However, the absence of a statistically significant causal effect of serum 25OHD on eBMD in women, regardless of menopausal status, suggests that other factors (ie, physical activity), may have a greater effect on female eBMD, which underscores the need for further investigation into sex-specific mechanisms influencing bone health. Additionally, our study suggests the positive causal effect of serum 25OHD levels on eBMD in individuals aged 65 years and older. Mechanistically, decreased serum 25OHD levels negatively affect bone metabolism by reducing calcium absorption and increasing parathyroid hormone production (43). With advancing age, these effects may be exacerbated due to declined metabolic function, and an increased vulnerability to compromised bone health, especially in those aged 65 years and older.

In contrast to the limited causal evidence observed, our cross-trait meta-analysis revealed multiple horizontal pleiotropic signals. Among 49 pleiotropic loci, 9 showed strong evidence for sharing a common causal variant (PPH4 > 0.75). Here, we highlight 2 interesting examples of colocalized loci, *RPS9* and *RPL7A*, both of which were also identified as hub genes in the total PPI network. The gene *RPS9* encodes a ribosomal protein that plays a role in translational elongation, including messenger RNA unwinding and decoding accuracy (44). *RPL7A* is a component of the 60S ribosomal subunit, which is involved in cell growth and differentiation (45). Both genes have been implicated in the potential development of osteosarcoma (44, 45). At first glance, osteosarcoma is a primary bone tumor that mainly affects adolescents and young adults (44), while osteoporosis is a chronic metabolic bone disease that mainly affects postmenopausal women and older adults (1). Nevertheless, it is crucial to recognize that bone is commonly affected in tumors, where bone loss may result from tumor-derived circulating hormones and cytokines that compromise local bone formation (46). We therefore suggest that the effect of these genes on bone health be investigated in more detail.

One advantage of a cross-trait meta-analysis is that combining association evidence across multiple studies can reveal signals that might not reach genome-wide significance in a single-trait analysis. Indeed, we identified 4 novel loci shared between serum 25OHD and eBMD. Here, we emphasize 2 intriguing examples. First, *NFIX*, located at the most significant novel locus (index SNV: rs1077151), encodes CCAAT-box-binding transcription factor, which binds the palindromic sequence in viral and cellular promoter. Recent studies have found that overexpression of *NFIX* disrupts bone homeostasis by positively regulating osteoclast differentiation and negatively regulating osteoblast differentiation (47). This dual regulatory role suggests a potential effect on BMD, as an imbalance between osteoclast and osteoblast activities can lead to bone resorption and reduced BMD. Second, *SLC2A4RG* and *ZGPAT* were mapped by the index SNV rs79873740, representing the only case where the locus was both novel and colocalized. *SLC2A4RG* has been identified as a gene preferentially mutated and copy number–altered in metastatic breast cancer (48). Given that bone is a common site for breast cancer metastasis, particularly at advanced stages (49), *SLC2A4RG* may play a role in bone integrity and, by extension, BMD. *ZGPAT* is a transcriptional repressor that negatively regulates the expression of epidermal growth factor receptor (*EGFR*) expression. It is noteworthy that the *EGFR* signaling pathway has an important regulatory role in bone function, primarily exerting an anabolic influence on bone metabolism, which can subsequently affect BMD (50). While direct evidence linking these genes to serum 25OHD levels is limited, their effect on bone metabolism suggests they could indirectly affect vitamin D utilization or requirements in bone remodeling processes. Follow-up experimental studies are warranted to provide more detailed functional annotation in the serum 25OHD-eBMD relationship.

At the gene-tissue pair level, TWAS results further revealed shared mechanistic hypotheses by leveraging GWAS and GTEx statistics. The 6 loci identified both in TWAS and colocalization analysis implicate common biological mechanisms in serum 25OHD and eBMD regulation, involving cell-cycle and epigenetic regulation, protein synthesis and degradation, cell signaling, and immune response (40). In addition to digestive and exocrine/endocrine systems (well-established as relevant to serum 25OHD and BMD), we also revealed shared regulatory features in the nervous and cardiovascular systems, suggesting the possibility of shared pathways extending to a wider range of organs. Previous research has identified vitamin D deficiency as a common thread in the increased risks of multiple neurodegenerative and psychiatric disorders, possibly via regulating inflammatory mediators (51). Meanwhile, individuals with low BMD may experience structural changes in the cerebral cortex, suggesting the presence of the bone-brain axis (52). As another major cause of mortality and morbidity, cardiovascular diseases are linked with both traits. Maintaining optimal vitamin D levels has potential benefits in preventing cardiovascular events (53), while low BMD may independently predict cardiovascular mortality (54). Further studies are needed to elucidate the role of these hypothesized mechanisms.

Our findings hold important translational and clinical implications. First, in light of positive causal effects of serum 25OHD on eBMD among men and older adults, our findings underscore the importance of prioritizing vitamin D supplementation, particularly within specific demographics (men and older adults aged ≥65 years), to enhance BMD and prevent osteoporosis. However, for other demographic groups, ensuring consumption of vitamin D at the recommended reference nutrient intake of 10 µg/d, along with maintaining appropriate serum 25OHD levels (≥50 nmol/L), is deemed sufficient (2). Second, our study identified a multitude of loci and genes shared between serum 25OHD and eBMD, facilitating an understanding of the intricate biological mechanisms. They may serve as alternative or novel therapeutic targets for osteoporosis, providing a fundamental basis for future experimental studies. These genetic factors not only affect serum 25OHD and eBMD, but are also associated with other health factors (ie, tumor factors, neurological conditions, and cardiovascular health), which suggests their pivotal role in tailoring treatments to individuals and advancing comprehensive health management approaches.

Several limitations need to be acknowledged. First, to avoid potential bias from population stratification, our study exclusively considered genetic data of European ancestry, which constrains the applicability of the findings to other ethnic populations. While the one-sample MR suggested a causal effect in men and older adults aged 65 years and older, we were unable to perform other analyses (ie, cross-trait meta-analysis and TWAS) stratified by these subgroups due to the unavailability of specific GWAS summary data. Second, both serum 25OHD and eBMD GWASs contained UKB individuals, which may introduce bias due to sample overlap, especially for MR analysis (55). We estimated such a potential bias as previously described (55) and found that the bias due to sample overlap was estimated to be as 0.002, while the expected type I error rate was 0.06, suggesting minimal influence. We also conducted sensitivity analysis by including DXA-derived BMDs without UKB individuals, as secondary outcomes. The results consistently paralleled the main analysis results, affirming a negligible influence of sample overlap. Furthermore, we identified the genes and tissues relevant to serum 25OHD and eBMD solely based on functional data sets and algorithms. In-depth experimental studies are needed to elucidate the mechanisms.

To conclude, leveraging a well-established genome-wide cross-trait framework, we extend our understanding of the genetic overlap between serum 25OHD and eBMD. Our study supports a positive causal association between serum 25OHD and eBMD in men and individuals aged 65 years and older. Also, we identified multiple pleiotropic loci and shared gene-tissue pairs that may potentially be targeted for further investigation. Overall, these findings reinforce the idea that 25OHD and eBMD implicate shared common biological processes and open new avenues for future molecular and functional validation, disease prevention, and clinical treatment of osteoporosis.

## Data Availability

Data from the UKB are available for researchers. Full information on how to access data can be found at the website (https://www.ukbiobank.ac.uk/enable-your-research). GWAS data sets of serum 25OHD are accessible from the Program in Complex Trait Genomics website (https://cnsgenomics.com/content/data). GWAS data sets for BMD traits are accessible from the GEnetic Factors for OSteoporosis Consortium website (GEFOS, http://www.gefos.org/)

## Abbreviations

25OHD: 25-hydroxyvitamin D
BMD: bone mineral density
BMI: body mass index
DXA: dual-energy x-ray absorptiometry
eBMD: estimated heel bone mineral density
GWAS: genome-wide association study
IVs: instrumental variables
IVW: inverse-variance weighted
LD: linkage disequilibrium
LDSC: linkage disequilibrium score regression
MR: mendelian randomization
PLEIO: Pleiotropic Locus Exploration and Interpretation using Optimal test
PPI: protein-protein interaction
SNV: single-nucleotide variation
STRING: Search Tool for the Retrieval of Interacting Genes/Proteins
SUPERGNOVA: SUPER GeNetic cOVariance Analyzer
TWAS: transcriptome-wide association studies
UKB: UK Biobank
